# Clinical Importance of Angiogenic Cytokines, Fibrinolytic Activity and Effusion Size in Parapneumonic Effusions

**DOI:** 10.1371/journal.pone.0053169

**Published:** 2013-01-07

**Authors:** Chi-Li Chung, Shih-Hsin Hsiao, George Hsiao, Joen-Rong Sheu, Wei-Lin Chen, Shi-Chuan Chang

**Affiliations:** 1 Division of Pulmonary Medicine, Department of Internal Medicine, Taipei Medical University Hospital, Taipei, Taiwan; 2 School of Respiratory Therapy, College of Medicine, Taipei Medical University, Taipei, Taiwan; 3 Graduate Institute of Medical Sciences and Department of Pharmacology, College of Medicine, Taipei Medical University, Taipei, Taiwan; 4 Department of Chest Medicine, Taipei Veterans General Hospital, Taipei, Taiwan; 5 Institute of Emergency and Critical Care Medicine, National Yang-Ming University, Taipei, Taiwan; Statens Serum Institute, Denmark

## Abstract

**Objective:**

To investigate the relationship among angiogenic cytokines, fibrinolytic activity and effusion size in parapneumonic effusion (PPE) and their clinical importance.

**Methods:**

From January 2008 through December 2010, 26 uncomplicated (UPPE) and 38 complicated (CPPE) PPE were studied. Based on chest ultrasonography, there were non-loculated in 30, uni-loculated in 12, and multi-loculated effusions in 22 patients. The effusion size radiological scores, and effusion vascular endothelial growth factor (VEGF), interleukin (IL)-8, plasminogen activator inhibitor type-1 (PAI-1) and tissue type plasminogen activator (tPA) were measured on admission. Treatment outcome and pleural fibrosis, defined as radiological residual pleural thickening (RPT), were assessed at 6-month follow-up.

**Results:**

The effusion size and effusion VEGF, IL-8 and PAI-1/tPA ratio were significantly higher in CPPE than in UPPE, and significantly higher in multi-loculated PPE than in non-locualted and uni-loculated PPE, respectively. VEGF (cutoff value 1975 pg/ml) and IL-8 (cutoff value 1937 pg/ml) seemed best to discriminate between UPPE and CPPE. VEGF, IL-8 and effusion size correlated positively with PAI-1/tPA ratio in both UPPE and CPPE. Moreover, the level of VEGF, but not IL-8, correlated positively with effusion size in all patients (r = 0.79, p<0.001) and in UPPE (r = 0.64, p<0.001) and CPPE (r = 0.71, p<0.001) groups. The patients with higher VEGF or greater effusion were prone to have medical treatment failure (n = 10; VEGF, odds ratio 1.01, p = 0.02; effusion size, odds ratio 1.26, p = 0.01). Additionally, ten patients with RPT had larger effusion size and higher levels of VEGF and PAI-1/tPA ratio than did those without.

**Conclusions:**

In PPE, VEGF and IL-8 levels are valuable to identify CPPE, and higher VEGF level or larger effusion is associated with decreased fibrinolytic activity, development of pleural loculation and fibrosis, and higher risk of medical treatment failure.

## Introduction

Formation of parapneumonic effusion (PPE) involves increased vascular permeability of the pleura induced by the contiguous pneumonia. Exposure of pleural mesothelial cells to bacteria or lipopolysaccharide may increase release of angiogenic factors, including vascular endothelial growth factor (VEGF) and interleukin (IL)-8, induce vascular hyperpermeability and pleural fluid production, activate coagulation cascade, and repress fibrinolytic activity within the pleural cavity [1], [2], leading to the development of a “fibrinopurulent” or “complicated” PPE (CPPE) [3].

Fluid loculation with fibrin septation is commonly found to be the initial presentation of CPPE and associated with poor outcome [4], [5]. Fibrin turnover in the pleural cavity is affected by fibrinolytic activity mediated by plasmin, which is regulated by the equilibrium between plasminogen activators (PAs) and plasminogen activator inhibitors (PAIs) [6]. An imbalance between PAI-1 and tissue type plasminogen activator (tPA) may elicit fibrin formation and subsequent pleural fluid loculation and fibrosis [5], [7].

VEGF may facilitate the genesis of fibrin gel in PPE [8]. Previous studies reported that VEGF might play a role in the modulation of tPA and PAI-1 [9], and that anti-VEGF antibody could attenuate pleurodesis and reduced fluid volume of inflammatory pleural effusion in experimental models [10]–[12]. These findings suggest that VEGF may be involved in the regulation of fibrin turnover and fluid loculation in the pleural cavity and subsequent residual pleural thickening (RPT) or fibrosis [8]. However, the clinical relevance of angiogenic cytokines, fibrinolytic activity and effusion volume in PPE remains unclear. The aim of the present study was to evaluate the relationship among angiogenic cytokines (VEGF, IL-8), fibrinolytic parameters (tPA and PAI-1) and effusion size in PPE, and their clinical importance.

## Methods

### Study Design

This single-center, prospective study intended to assess the clinical importance of angiogenic cytokines, fibrinolytic activity and effusion size in PPE. Ethics approval (CRC-05-11-01) was obtained from the Institutional Review Board of Taipei Medical University (Taipei, Taiwan), and all patients gave written informed consent before entering the study.

### Patient Selection

Consecutive patients with pleural effusions (PE) of unknown causes admitted to Taipei Medical University Hospital were eligible for this study, and were included when a diagnosis of PPE was established. Exclusion criteria were as follows: history of invasive procedures directed into the pleural cavity; recent severe trauma, hemorrhage, or stroke; bleeding disorder or anticoagulant therapy; use of streptokinase in the previous 2 years.

### Imagings of PE

PE were evaluated and divided into loculated or non-loculated effusions by chest radiography (CXR), chest ultrasonography (US), or thoracic computed tomography (CT) scans as previously described [5]. Patients with loculated effusions were subdivided into uni-loculated and multi-loculated effusion groups by chest US. The patients with multiple loculi of effusions divided by fibrin septa were classified into multi-loculated effusion group, and those who had a single loculated effusion without fibrin septation were classified into uni-loculated effusion group (see Protocol S1) [13].

### CXR Scoring

The posteroanterior CXR films were read and scored by two radiologists who were blind to any clinical information to determine (a) the largest linear width of pleural opacity and (b) effusion size CXR score: the estimated overall percentage of pleural shadowing in the hemithorax (see Protocol S2) [14].

### Thoracentesis and Pleural Fluid Analysis

With the guidance of chest US, 50 ml of pleural fluid was aspirated immediately or within 24 hours after hospitalization. When PE was multi-loculated, the fluid was aspirated from the largest loculus. Pleural fluid analyses and microbiological studies were performed routinely.

### Measurement of Effusion VEGF, IL-8, PAI-1 and tPA

The commercially available enzyme-linked immunosorbent assay kits were used to measure effusion levels of VEGF, IL-8 (R & D System; Minneapolis, MN, USA), tPA and PAI-1 (American Diagnostica; Greenwich, CT, USA) as previously described [5].

### Management of PPE

All patients initially received empiric broad-spectrum antibiotics, which were appropriately adjusted later based on the results of microbiological studies and clinical response. PPE were classified as uncomplicated PPE (UPPE) or CPPE. CPPE was defined as a PPE with one of the following criteria: (1) pH<7.2; (2) glucose<60 mg/dl; (3) LDH>1000 U/l; (4) bacteria found on Gram’s stain or culture; (5) frank pus [15], [16]. A 10–14F pigtail tube was inserted into the largest loculus with US guidance to drain the effusion once a CPPE was diagnosed. Clinical response was assessed 24 hours later, and the patients who met all the following criteria: (a) loculated effusion; (b) less than 50% improvement in effusion CXR score after initial drainage; (c) persistent fever (>38°C) or dyspnea (respiratory rate >20/min), were subjected to intrapleural injection with streptokinase (IPSK) 250000 IU once daily for 3 days [14]. After injection, the pigtail tube was clamped for two hours and then opened for free drainage.

### Outcome Measures

Treatment response was assessed from day 1 (start of antibiotic treatment) to day 5 by (a) vital signs; (b) complete blood count; (c) CXR; and (d) volume of effusion drained (for CPPE patients). For UPPE patients, those who had improvement in vital signs and pleural opacity on CXR were defined as *medical success*, whereas those having progressive sepsis and enlarging pleural opacity were re-classified and treated as CPPE group. For CPPE patients, the pigtail tube was removed for those treated with *medical success* when the drainage was less than 50 ml in the last 24 hours, whereas those who had both (a) ongoing or progressive sepsis syndrome [17] and (b) less than 50% reduction in pleural opacity on CXR beyond 5 days after pigtail drainage were defined as *medical failure* and subjected to surgical intervention if clinically indicated (see Protocol S3 for definitions of *medical success and medical failure*) [18].

CXR and pulmonary function testing with spirometry were performed on discharge and 6 months later, respectively. RPT was measured and defined as a lateral pleural thickening of ≥10 mm shown on CXR and confirmed by chest US or CT at the end of 6-month follow-up [19].

### Statistical Analysis

Data were expressed as mean ± SD, median (interquartile range or range) or frequency (%), where appropriate. Comparisons of continuous data were made using an unpaired *t* test or Mann–Whitney U test between two groups, and one-way analysis of variance with *post hoc* Duncan test or Kruskal-Wallis test with *post hoc* Dunn’s test among three groups, where appropriate. The optimal sensitivity, specificity and cutoff value of pleural fluid variables for distinguishing UPPE from CPPE were evaluated by the receiver operating characteristics by analyzing the area under the curve (AUC). The correlations between variables were determined by Spearman rank correlation coefficients. Categorical variables between two groups were examined using χ^2^ method and/or Fisher’s exact test, when appropriate. A two-tailed p value <0.05 was considered to be statistically significant.

Multivariate logistic regression analyses were performed to determine factors independently associated with *medical failure*, as compared with patients with *medical success*. Variables found to be significant in the univariate analysis were entered into a binary logistic regression analysis. Results of multivariable analyses are reported as odds ratios with 95% confidence intervals and p-values.

## Results

### Patient Characteristics

Consecutive 72 patients with PPE were eligible for this study. Eight patients were excluded because of recent stroke in two, recent gastrointestinal bleeding in one and informed consent unavailable in five cases, respectively. Finally, 64 patients were enrolled, including 46 men and 18 women with an age range from 41 to 87 years (mean age, 64 years), and 60 of them completed 6 months of follow-up from January 2008 through December 2010.

### Comparisons between UPPE and CPPE

There were 26 patients with UPPE and 38 patients with CPPE. Clinical data (see Table S1), pleural fluid characteristics, angiogenic cytokines and parameters related to fibrinolytic activities in pleural fluids are shown in Table 1. Compared to patients with UPPE, CPPE patients were significantly younger and had significantly higher effusion CXR score on admission. No significant differences between the two groups were found in terms of gender, comorbidities, and duration of illness before treatment. Patients with CPPE had significantly higher levels of effusion VEGF, IL-8, PAI-1 and PAI-1/tPA ratio and lower values of tPA than did UPPE patients. Moreover, compared to UPPE, there was no significant increase in protein concentrations in CPPE.

**Table 1:** Pleural Effusion Size and Variables between Uncomplicated and Complicated Parapneumonic Effusions.

	All Patients	UPPE	CPPE	
	(n = 64)	(n = 26)	(n = 38)	p value†
**Effusion CXR score, %, mean ± SD**	48±14	38±8	55±14	<0.001
**pH value**	7.30	7.39	7.18	<0.001
	(7.19–7.41, n = 60)	(7.37–7.41, n = 26)	(6.92–7.39, n = 34)	
**Glucose, mg/dl**	108	123	69	<0.001
	(59–147)	(109–151)	(35–120)	
**Protein, g/l**	4.1	3.7	4.2	0.2
	(3.5–4.8)	(3.5–4.7)	(3.9–4.8)	
**LDH, IU/dl**	462	129	1622	<0.001
	(160–1808)	(97–258)	(566–3332)	
**Leukocyte count, cells/µl**	2805	1200	9660	0.003
	(520–11140)	(363–3410)	(1750–14175)	
**PAI-1, ng/ml**	99.9	53.0	104.0	<0.001
	(52.8–114.5)	(16.7–104.1)	(69.2–198.0)	
**tPA, ng/ml**	14.5	17.6	8.3	<0.001
	(5.8–20.0)	(14.0–21.0)	(3.3–17.0)	
**PAI-1/tPA ratio**	5.7	2.4	14.8	<0.001
	(3.0–17.7)	(1.1–5.1)	(5.5–29.0)	
**IL-8, pg/ml**	5377	175	6184	<0.001
	(194–6281)	(61–537)	(5906–6572)	
**VEGF, pg/ml**	2928	477	5255	<0.001
	(589–5266)	(185–1099)	(4792–5601)	

*Definition of abbreviations:* UPPE = uncomplicated parapneumonic effusion; CPPE = complicated parapneumonic effusion; Effusion CXR score = portion of hemithorax opacified by pleural effusion on posteroanterior chest radiograph; LDH = lactate dehydrogenase; PAI-1 = plasminogen activator inhibitor-1; tPA = tissue type plasminogen activator; IL-8 = interleukin-8; VEGF = vascular endothelial growth factor.

Data are presented as median (IQR) unless specified.

† For comparisons between UPPE and CPPE groups.

### Comparisons between Non-loculated, Uni-loculated and Multi-loculated PPE

All 26 patients with UPPE and four with CPPE had non-loculated effusion (non-loculated group, n = 30). The remaining 34 CPPE patients had loculated effusions and were further divided into uni-loculated (n = 12) and multi-loculated (n = 22) groups (Table 2). Compared to patients with non-loculated and uni-loculated PPE groups, multi-loculated PPE patients had significantly higher effusion CXR score, lower levels of glucose and tPA, and higher values of VEGF, IL-8 and PAI-1/tPA ratio in the pleural fluids. Additionally, patients with uni-loculated PPE had significantly higher effusion CXR score, lower value of pH, and higher levels of LDH, VEGF, IL-8, PAI-1 and PAI-1/tPA ratio than did those with non-loculated PPE. However, there were no significant differences in protein concentrations among the three groups.

**Table 2:** Pleural Effusion Size and Variables between Non-, Uni- and Multi-loculated Parapneumonic Effusions (PPE).

	Non-loculated PPE†	Uni-loculated PPE	Multi-loculated PPE	
	(n = 30)	(n = 12)	(n = 22)	p value
**Effusion CXR score, %, mean ± SD**	39±9	48±16*	61±10** ^,^ #	<0.001
**pH value**	7.40	7.12***	7.15***	<0.001
	(7.37–7.41)	(6.92–7.19)	(6.80–7.30, n = 18)	
**Glucose, mg/dl**	123	111	57*** ^,^ ##	<0.001
	(108–163)	(60–157)	(27–90)	
**Protein, g/l**	3.7	4.4	4.2	0.24
	(3.5–4.7)	(2.0–5.0)	(4.0–4.8)	
**LDH, IU/dl**	155	636**	1677***	<0.001
	(97–320)	(300–2263)	(760–3332)	
**Leukocyte count, cells/µl**	1146	5155*	10080***	0.004
	(356–3410)	(2060–13221)	(1750–19710)	
**PAI-1, ng/ml**	53.0	108.1**	104.0***	<0.001
	(16.7–104.1)	(69.2–198.0)	(92.4–212.0)	
**tPA, ng/ml**	15.0	15.1	8.3* ^,^ #	0.03
	(8.7–21.0)	(4.8–25.9)	(3.2–16.7)	
**PAI-1/tPA ratio**	3.5	11.4**	16.1*** ^,^ #	<0.001
	(1.1–5.9)	(4.4–21.3)	(10.1–32.3)	
**IL-8, pg/ml**	193	5905***	6484*** ^,^ #	<0.001
	(61–670)	(4586–6348)	(6009–6773)	
**VEGF, pg/ml**	530	2928***	5384*** ^,^ ###	<0.001
	(185–1645)	(1861–4887)	(5255–5601)	

See Table 1 for definition of the abbreviations.

Data are presented as the median (IQR) unless specified.

† Non-loculated PPE group includes 26 UPPE and 4 CPPE patients.

* p<0.05,

** p<0.01 and

*** :p<0.001 *versus* non-loculated PPE group;

# p<0.05,

## p<0.01 and

### :p<0.001 *versus* uni-loculated PPE group**.**

### Optimal Sensitivity, Specificity and Cutoff Value of Pleural Effusion Size and Variables for the Identification of CPPE

Among the biochemical parameters, LDH>1019 IU/dl had best sensitivity (84%) and specificity (100%) to identify CPPE, with AUC of 0.97 (Table 3). In contrast, pH value, glucose and leukocyte count had relatively lower sensitivity in differentiating CPPE from UPPE.

**Table 3:** Optimal Sensitivity, Specificity and Cutoff Value of Pleural Effusion Size and Variables for the Diagnosis of Complicated Parapneumonic Effusions.

	Cutoff	Sensitivity, %	Specificity, %	AUC	95% CI
**Effusion CXR score**	>47%	74	92	0.85	0.73–0.92
**pH value**	≤7.19	75	100	0.87	0.76–0.94
**Glucose**	≤57 mg/dl	68	100	0.77	0.65–0.87
**LDH**	>1019 IU/dl	84	100	0.97	0.89–0.99
**Leukocyte count**	>6000 cells/µl	58	92	0.77	0.65–0.87
**PAI-1**	>53 ng/ml	90	62	0.78	0.66–0.88
**tPA**	≤12.7 ng/ml	63	85	0.75	0.62–0.85
**PAI-1/tPA ratio**	>5.9	74	92	0.90	0.80–0.96
**IL-8**	>1937 pg/ml	95	100	0.99	0.93–1.00
**VEGF**	>1975 pg/ml	90	100	0.99	0.92–1.00

See Table 1 for definition of the abbreviations. AUC = area under the curve; CI = confidence interval.

Among the pleural fluid variables, IL-8 at the cutoff level >1937 pg/ml had highest sensitivity and specificity to discriminate between UPPE and CPPE (AUC, 0.99; sensitivity, 95%; specificity, 100%), followed by VEGF>1975 pg/m (AUC, 0.99; sensitivity, 90%; specificity, 100%), PAI-1/tPA ratio5.9 (AUC, 0.90; sensitivity, 74%; specificity, 92%) and effusion CXR score >47% (AUC, 0.85; sensitivity, 74%; specificity, 92%) (Table 3).

### Correlations among Effusion Angiogenic Cytokines, Fibrinolytic Parameters, Pleural Fluid Characteristics and Effusion CXR Score

As shown in Table 4, the effusion levels of IL-8 and VEGF were positively correlated with those of LDH, leukocyte count, and PAI-1/tPA ratio, and negatively correlated with those of pH and glucose in both UPPE and CPPE. In addition, VEGF correlated positively with PAI-1 and negatively with tPA in CPPE.

**Table 4:** Correlation among Angiogenic Cytokines, Fibrinolytic Parameters, Pleural Fluid Characteristics and Effusion CXR scores.

	pH	Glucose	LDH	Leukocyte count	PAI-1	tPA	PAI-1/tPA ratio	Effusion CXR score
**UPPE (n = 26)**								
IL-8	−0.65‡	−0.47†	0.79‡	0.42†	0.31	− 0.21	0.42†	0.09
VEGF	−0.41*	−0.35*	0.57‡	0.36*	0.27	−0.24	0.49‡	0.64‡
Effusion CXR score	−0.25	0.25	−0.08	0.10	0.51†	0.05	0.44*	−
**CPPE (n = 38)**								
IL-8	−0.53‡	−0.37†	0.48‡	0.52‡	0.24	−0.36†	0.50‡	0.32
VEGF	−0.57‡	−0.64‡	0.65‡	0.46†	0.49‡	−0.43†	0.59‡	0.71‡
Effusion CXR score	−0.32	−0.26	0.29	−0.20	0.26	−0.44†	0.51‡	−

See Table 1 for definition of the abbreviations.

* Correlation is statistically significant at the level of 0.05.

† Correlation is statistically significant at the level of 0.01.

‡ Correlation is statistically significant at the level of 0.001.

The effusion CXR score had significant positive correlation with the effusion levels of VEGF and PAI-1/tPA ratio in both UPPE and CPPE groups (Table 4) as well as in all patients (VEGF, r = 0.79, p<0.001; PAI-1/tPA ratio, r = 0.59, p<0.001, respectively). However, there was no significant correlation between the effusion size and the effusion levels of IL-8.

### Managements of PPE and Treatment Response

All 38 CPPE patients required effusion drainage in addition to antibiotic treatments. After initial drainage for 24 hours, five uni-loculated and 18 multi-loculated CPPE patients who had ongoing fever or dyspnea and less than 50% improvement in effusion CXR score, underwent IPSK therapy. However, 10 multi-loculated CPPE patients who failed to improve after IPSK therapy and had progressive sepsis and insignificant radiological improvement beyond 5 days of pleural drainage, were classified as *medical failure* group and subjected to video-assisted thoracoscopic surgery (VATS) decortication. In contrast, the remaining 28 CPPE patients who showed remarkable improvement after medical treatment (effusion drainage with or without IPSK) were designated as *medical success* group. In addition, all 26 UPPE patients who responded well to antibiotic treatment alone were included into *medical success* group as well (Table 5).

**Table 5:** Pleural Effusion Status and Variables between Medical Treatment Success and Medical Treatment Failure Patients.

	Medical success	Medical failure	
	(n = 54)	(n = 10)	p value
**Effusion status**			
Effusion CXR score, %, mean ± SD	45±13	66±6	<0.001
Multi-loculation, n (%)	12 (22)	10 (100)	<0.001
**Pleural fluid**			
pH value	7.23 (7.15–7.41, n = 53)	7.14 (6.80–7.3, n = 7)	0.16
Glucose, mg/dl	116 (77–157)	51 (27–90)	<0.001
LDH, IU/dl	316 (129–1618)	2219 (1519–3332)	<0.001
Leukocyte count, cells/µl	2080 (460–8230)	10080 (1450–185000)	0.09
PAI-1, ng/ml	104.0 (80.8–198.0)	102.5 (52.6–117.0)	0.46
tPA, ng/ml	11.5 (4.2–17.4)	8.2 (3.2–17.0)	0.16
PAI-1/tPA ratio	13.6 (4.5–26.9)	13.1 (5.5–17.1)	0.10
IL-8, pg/ml	6141 (4845–6349)	6349 (6122–6773)	<0.001
VEGF, pg/ml	3872 (2019–5271)	5503 (5384–5601)	<0.001

See Table 1 for definition of the abbreviations. Data are presented as the median (IQR) unless specified.

### Comparisons between *Medical Success* and *Medical Failure* Groups

As shown in Table 5, the effusion CXR score on admission and the occurrence of multi-loculated effusion were significantly higher in medical failure group than in medical success group. Moreover, medical failure patients had significantly higher effusion levels of LDH, IL-8 and VEGF, and lower level of glucose than did those with medical success. However, the effusion values of PAI-1, tPA and PAI-1/tPA ratio showed no significant differences between the two groups.

### Multivariate Logistic Regression Analysis

Furthermore, multivariate logistic regression analysis was used to identify the independent risk factor for *medical failure* (Table 6). Variables of significance in univariate analysis were included and VEGF and effusion CXR score were treated separately because they were mutually correlated. As a result, only higher effusion VEGF level or greater effusion CXR score was an independent risk factor for *medical failure*.

**Table 6:** Multivariate Logistic Regression Analyses of Factors Associated with Medical Treatment Failure.

	VEGF excluded			Effusion CXR score excluded		
	Odds ratio	95% CI	p value	Odds ratio	95% CI	p value
**Effusion status**						
Effusion CXR score, %	1.26	1.06–1.51	0.01			
Multi-loculation	1.00	0.99–1.00	0.99	1.00	0.99–1.01	0.99
**Pleural fluid**						
Glucose, mg/dl	1.01	0.98–1.04	0.52	0.99	0.97–1.03	0.76
LDH, IU/dl	1.00	0.99–1.00	0.87	1.00	0.99–1.00	0.99
IL-8, pg/ml	1.00	0.99–1.00	0.93	1.00	0.99–1.00	1.00
VEGF, pg/ml				1.01	1.00–1.02	0.02

See Table 1 for definition of the abbreviations. CI = confidence interval.

### Follow-up Period

All 64 patients were successfully treated and discharged uneventfully. Sixty patients were followed up regularly for 6 months. Either assessed on discharge or at 6-month follow-up, the effusion CXR score and effusion thickness were significantly greater, and the forced vital capacity (FVC) were significantly lower in CPPE patients than in UPPE patients (see Table S2).

### Comparisons between PPE Patients with and without RPT

RPT was observed in 10 of 60 (16.7%) patients who completed 6-month follow-up (Table 7). Most of them (80%) had multi-loculation of pleural effusions initially. The effusion CXR score on admission and the effusion levels of PAI-1, PAI-1/tPA ratio and VEGF, but not IL-8, were significantly higher, and the FVC was significantly lower in the patients with RPT than in those without.

**Table 7:** Pleural Fluid Variables and Pulmonary Function in Patients With or Without Development of Residual Pleural Thickening (RPT).

	RPT (−)	RPT (+)	
	(n = 50)	(n = 10)	p value
**Effusion status**			
Effusion CXR score, %, mean ± SD	46±14	61±11	0.005
Loculation, n (%)	23 (46)	10 (100)	0.001
Multi-loculation, n (%)	14 (28)	8 (80)	0.002
**Pleural fluid**			
PAI-1, ng/ml	99.9 (53.0–110.7)	212.0 (92.4–258.0)	0.04
tPA, ng/ml	15.8 (4.8–21.0)	10.4 (8.2–12.7)	0.08
PAI-1/tPA ratio	5.2 (2.4–14.8)	17.1 (14.1–24.8)	0.006
IL-8, pg/ml	3262 (193–6348)	6122 (5906–6213)	0.06
VEGF, pg/ml	1975 (530–5186)	5384 (5271–5601)	<0.001
**FVC, % predicted**			
At 6 months	79 (78–80)	75 (74–76)	<0.001

See Table 1 for definition of the abbreviations. RPT = residual pleural thickening ≥10 mm shown on CXR at the end of 6-month follow-up; FVC = forced vital capacity. Data are presented as median (IQR) unless specified.

## Discussion

Our results demonstrated that effusion size reflected by radiological scores and effusion levels of VEGF, IL-8 and PAI-1/tPA ratio were significantly higher in CPPE than in UPPE, and significantly higher in multi-loculated PPE than in non-loculated and uni-loculated PPE, respectively. VEGF (cutoff value, 1975 pg/ml) and IL-8 (cutoff value, 1937 pg/ml) seemed best to discriminate between UPPE and CPPE. VEGF, IL-8 and effusion size correlated positively with PAI-1/tPA ratio, and VEGF, but not IL-8, had significant positive correlation with effusion size in all patients and in both UPPE and CPPE groups. Patients with higher effusion VEGF level or greater effusion size were prone to have medical treatment failure. Ten patients with RPT had larger effusion size and higher levels of VEGF and PAI-1/tPA ratio than did those without. To our knowledge, this is the first study to demonstrate that effusion angiogenic cytokines correlated significantly with pleural fibrinolytic activity, and that the elevated VEGF level or effusion size was associated with poor outcome in PPE.

Previous studies showed that the level of VEGF was consistently higher in exudative than in transudative pleural effusions [1], [20], [21], and empyema fluids contained significantly higher levels of VEGF than did UPPE [22]. VEGF induces extravascular leakage of plasma proteins and is important in the modulation of extracellular matrix proteolysis by regulating the expression of tPA and PAI-1 in endothelial cells [9]. Furthermore, VEGF has been reported to increase PAI-1 expression in keloid fibroblasts and to contribute to dermal fibrosis [23]. Another angiogenic factor IL-8 has been shown to increase vascular permeability and fluid exudation in endotoxin- induced pleurisy *in vivo* [24], to correlated positively with PAI-1 and negatively with tPA in exudative PE [25], and might hold an important role in successful pleurodesis [26]. All these findings suggest that angiogenic cytokines may elicit exudative effusions and modulate fibrinolytic activity in pleural space by altering the balance of PAI-1 and tPA.

In agreement with previous report by Marchi et al [27], the present study showed that effusion levels of VEGF and IL-8 were significantly higher in CPPE than in UPPE and were positively correlated with LDH values. However, Marchi et at failed to demonstrate the superiority of effusion VEGF and IL in distinguishing CPPE from UPPE as compared with effusion glucose and LDH. At variance with the results of the previous study [27], our results indicated that IL-8>1937 pg/ml or VEGF>1975 pg/ml had a better sensitivity and specificity to identify CPPE than did convention biochemical markers. The discrepancy might be due to the different definition of CPPE used in the present study [15], [16], which seemed more stringent than that of the previous report [27]. The usefulness of effusion angiogenic cytokines in determining CPPE may be limited in usual clinical practice, since measuring cytokines is time consuming. Nevertheless, our results indicated that VEGF and IL-8 might play an important role in the evolution of UPPE to CPPE.

Furthermore, we investigated the relationship between angiogenic cytokines and fibrinolytic parameters and demonstrated that the levels of VEGF and IL-8 positively correlated with the values of PAI-1/tPA ratio in both UPPE and CPPE, though only VEGF levels correlated positively with PAI-1 values and negatively with tPA values in CPPE. In addition, the levels of VEGF, IL-8, PAI-1 and PAI-1/tPA ratio were significantly higher and the values of tPA were significantly lower in multi-loculated than in non-loculated and uni-loculated PPE. These findings are in keeping with the results of the previous *in vitro* study [9], and raise the possibility that angiogenic cytokines, particularly VEGF, may attenuate pleural fibrinolytic activity by disrupting the balance of PAI-1 and tPA elaborated by endothelial and/or mesothelial cells in PPE. Moreover, our results showed that effusion CXR scores positively correlated with the levels of VEGF and PAI-1/tPA ratio, but not IL-8, in both UPPE and CPPE. These findings suggest that the effusion size on chest radiograph may indirectly reflect both the angiogenic and fibrinolytic activities of the underlying pleural fluids and that the increase in VEGF is associated with the decrease in fibrinolytic activity and subsequent fibrin deposition and fluid loculation.

The predictors affecting the outcome of medical treatment in patients with PPE remain elusive. Two previous studies reported that loculation and effusion size were not related to clinical outcome of CPPE patients receiving medical therapy [28], [29]. On the contrary, other reports suggested that effusion drainage might be failed when effusion size was >40% of the hemithorax [30], and that pleural fluid loculation was a predicting factor for poor outcome of tube drainage for CPPE [4]. In our study, the results of multivariate analysis demonstrated that larger effusion size or higher effusion level of VEGF was the independent risk factor for failure of medical treatment, suggesting that the enhanced vascular permeability with increased pleural fluid exudation might impair medical therapy in PPE. Moreover, at variance with the previous report [4], our study indicated that the presence of multi-loculation did not increase the risk of treatment failure. The discrepancy between our and previous studies may be explained in part by that all CPPE patients in the present study received chest US-guided drainage and streptokinase was administered for the patients with loculated effusions who failed to improve after the initial drainage, which may minimize the effect of effusion loculation on the treatment outcome.

In this study, ten patients who developed RPT at the end of follow-up presented initially with loculated CPPE, and had greater effusion size and higher effusion levels of VEGF, PAI-1 and PAI-1/tPA ratio. A previous *in?vivo* study demonstrated that angiogenesis was required in the development of pleural fibrosis [10], [11]. A recent study showed that RPT was related to the pleural fluid VEGF levels in patients with PPE [31]. In agreement with the previous reports [10], [31], our study indicated that the increased angiogenic activity in the pleural fluid might contribute to subsequent development of pleural fibrosis in PPE. Furthermore, our results signified the possible role of VEGF-related impaired fibrinolytic activity in the formation of RPT in PPE and that the patients with RPT had a significantly lower FVC than did those without.

The benefit of intrapleural fibrinolytic agents in treating CPPE remains controversial. The largest randomized trial to date on the use of intrapleural streptokinase for treating CPPE and empyema (MIST1) could not demonstrate the efficacy of this treatment modality [32]. However, a meta-analysis of seven randomized controlled trials, including the MIST1, concluded that intrapleural fibrinolytic therapy (streptokinase or urokinase) conferred significant benefit in reducing the requirement for surgical intervention in patients who had either loculation or empyema [33]. Furthermore, a recently published randomized trial (MIST2) showed that a combination of intrapleural tPA and DNase significantly increased the drainage of pleural fluid and reduced the need of surgical referral and the length of hospital stay [34], which might pertain to our findings of significantly lower tPA levels in multi-loculated PPE and suggested a potential benefit of fibrinolytics in the management of loculated PPE.

In conclusion, the present study indicated that the increased levels of angiogenic cytokines were associated with decreased fibrinolytic activity in PPE. Higher levels of VEGF correlate with larger effusion size and PAI-1/tPA imbalance, contribute to fluid loculation and residual fibrosis, and appear to be a factor independently associated with medical treatment failure. Further large-scale studies are warranted to investigate the clinical usefulness of effusion VEGF level or effusion size score to predict treatment outcome in PPE patients, and more *in vitro* and *in vivo* experiments are required to clarify the causal relationships between effusion angiogenic cytokines and the genesis of pleural fibrins and fibrosis.

## Supporting Information

Table S1 **Demographic and Clinical Data of the Patients Studied.**
(DOCX)

Table S2 Comparisons of the Data on Discharge and at 6-month Follow-up* between the Patients with UPPE and CPPE.
(DOCX)

Protocol S1 **Criteria and validation for loculated and non-loculated effusions.**
(DOCX)

Protocol S2 **CXR scoring and validation.**
(DOCX)

Protocol S3 **Outcome measures for CPPE (complicated parapneumonic effusion) patients.**
(DOCX)
