# Supplementary material for: Clinical Importance of Angiogenic Cytokines, Fibrinolytic Activity and Effusion Size in Parapneumonic Effusions
Source: PLoS One. 2013 Jan 7;8(1):e53169. doi: 10.1371/journal.pone.0053169 (PMC3538784; doi:10.1371/journal.pone.0053169)
Supplement: Table S1 — Demographic and Clinical Data of the Patients Studied. (DOCX) [file pone.0053169.s001.docx]

**Table S1.** Demographic and Clinical Data of the Patients Studied

|  | **All Patients** | **UPPE** | **CPPE** |  |
| --- | --- | --- | --- | --- |
|  | **(n = 64)** | **(n = 26)** | **(n = 38)** | **p value^†^** |
| **Male, n (%)** | 46 (72) | 16 (62) | 30 (79) | 0.13 |
| **Age, yrs, mean ± SD** | 64 ± 15 | 72 ± 13 | 61 ± 14 | 0.002 |
| **Patients with comorbidities, n (%) *** | 40 (63) | 18 (69) | 22 (58) | 0.36 |
| **Symptom onset to treatment, days, median (range)** | 7 (5-11) | 7 (6-11) | 7 (5-9) | 0.19 |
| **Side of pleural effusion** |  |  |  |  |
| Right/left, n (%) | 46/18 (72/28) | 18/8 (69/31) | 28/10 (74/26) | 0.70 |
| **Status of pleural effusion** |  |  |  |  |
| Loculated/non-loculated, n (%) | 34/30 (53/47) | 0/26 (0/100) | 34/4 (89/11) | < 0.001 |

*Definition of abbreviations:* UPPE = uncomplicated parapneumonic effusion; CPPE = complicated parapneumonic effusion;

***** Comorbidities including alcoholism, diabetes mellitus, neurologic, cardiac, respiratory, liver and kidney diseases.

^†^ For comparisons between UPPE and CPPE groups.
