# Supplementary material for: Clinical Importance of Angiogenic Cytokines, Fibrinolytic Activity and Effusion Size in Parapneumonic Effusions
Source: PLoS One. 2013 Jan 7;8(1):e53169. doi: 10.1371/journal.pone.0053169 (PMC3538784; doi:10.1371/journal.pone.0053169)
Supplement: Table S2 — Comparisons of the Data on Discharge and at 6-month Follow-up* between the Patients with UPPE and CPPE. (DOCX) [file pone.0053169.s002.docx]

**Table S2.** Comparisons of the Data on Discharge and at 6-month Follow-up* between the Patients with UPPE and CPPE

|  | **All Patients** | **UPPE** | **CPPE** |  |
| --- | --- | --- | --- | --- |
|  | **(n = 64)** | **(n = 26)** | **(n = 38)** | **p Value**^†^ |
| **Length of hospital stay, days, median (range)** | 9 (6-20) | 7 (6-9) | 10 (8-20) | < 0.001 |
| **Follow up** |  |  |  |  |
| **Effusion CXR score, %, mean ± SD** |  |  |  |  |
| On discharge | 13 ± 9 | 6 ± 4 | 17 ± 10 | < 0.001 |
| At 6 months | 5 ± 3 | 2 ± 1 | 6 ± 5 | < 0.001 |
| **Lateral effusion thickness, mm, mean ± SD** |  |  |  |  |
| On discharge | 18 ± 13 | 9 ± 5 | 24 ± 14 | < 0.001 |
| At 6 months | 6 ± 5 | 2 ± 1 | 9 ± 7 | < 0.001 |
| **RPT, n (%)** | 10 (16) | 0 (0) | 10 (26) | 0.004 |
| **FVC, % predicted, median (IQR)** |  |  |  |  |
| On discharge | 74 (70-76) | 75 (74-78) | 72 (69-75) | < 0.001 |
| At 6 months | 78 (76-80) | 79 (78-80) | 78 (76-79) | 0.03 |

*Definition of abbreviations:* UPPE = uncomplicated parapneumonic effusion; CPPE = complicated parapneumonic effusion;

RPT = residual pleural thickening ≥ 10 mm shown on CXR at the end of 6-month follow-up; FVC = forced vital capacity.

*Sixty patients, including 23 UPPE and 37 CPPE patients, completed 6-month follow-up.

^†^ For comparisons between UPPE and CPPE groups.
