# Supplementary material for: Clinical Importance of Angiogenic Cytokines, Fibrinolytic Activity and Effusion Size in Parapneumonic Effusions
Source: PLoS One. 2013 Jan 7;8(1):e53169. doi: 10.1371/journal.pone.0053169 (PMC3538784; doi:10.1371/journal.pone.0053169)
Supplement: Protocol S1 — Criteria and validation for loculated and non-loculated effusions. (DOCX) [file pone.0053169.s003.docx]

**Protocol S1**

**Criteria and validation for loculated and non-loculated effusions**

All patients were subjected to routine chest radiography (frontal and lateral views), lateral decubitus view with the lesioned side down, and real-time chest US. Thoracic computed tomography (CT) was performed if clinically indicated. Loculated effusion was defined as an effusion with one or more of the following criteria: (1) failure of the effusion to gravitate to the most dependent part of the pleural spaces shown on lateral decubitus view of chest radiogram; (2) no significant change of the shape of fluid collection during deep breathing and/or panting on real-time chest US; (3) nondependent fluid collection, or fixed fluid collections with a convex inner margin seen on chest CT.

The chest US was performed by one of the two chest physicians who were blind to the clinical information of the patients. The sonograms were first interpreted by the examiner for determining loculated or non-loculated effusion, the presence of fibrinous strands and/or septation within pleural fluid, and associated pulmonary lesions. The sonograms were printed out and interpreted by another physician. When the two readers could not reach consensus, the case was presented to a third expert reader, and the adjudicated reading became final. In fact, no disagreement between the two readers on the determination of loculated or non-loculated effusion and on the presence of fibrin strands and/or septation was noted in this study.
