# Supplementary material for: Clinical Importance of Angiogenic Cytokines, Fibrinolytic Activity and Effusion Size in Parapneumonic Effusions
Source: PLoS One. 2013 Jan 7;8(1):e53169. doi: 10.1371/journal.pone.0053169 (PMC3538784; doi:10.1371/journal.pone.0053169)
Supplement: Protocol S2 — CXR scoring and validation. (DOCX) [file pone.0053169.s004.docx]

**Protocol S2**

**CXR scoring and validation**

Chest radiographs (CXRs) were saved as a digital image in the picture archiving and communication system (PACS) and the area occupied by the pleural opacity and the hemithorax were manually drawn around using the “pen tool”, forming a polyhedron exactly matching the area of interest. The area of each polyhedron (effusion and hemithorax) was measured using an image processing program provided by PACS. This permitted calculation of the percentage of hemithorax occupied by pleural effusion. Each CXR was measured by two independent radiologists who were blinded to any clinical information. When the differences in hemithorax or effusion area measurement were more than 5% between the two observers, they would have met and re-drawn the opacities together. In fact, there were no instances of disagreement between the observers working independently of >5% in the measured areas.
