# Supplementary material for: Clinical Importance of Angiogenic Cytokines, Fibrinolytic Activity and Effusion Size in Parapneumonic Effusions
Source: PLoS One. 2013 Jan 7;8(1):e53169. doi: 10.1371/journal.pone.0053169 (PMC3538784; doi:10.1371/journal.pone.0053169)
Supplement: Protocol S3 — Outcome measures for CPPE (complicated parapneumonic effusion) patients. (DOCX) [file pone.0053169.s005.docx]

**Protocol S3**

**Outcome measures for CPPE (complicated parapneumonic effusion) patients**

The clinical response to medical therapy (antibiotics and pigtail drainage with or without IPSK [intrapleural injection with streptokinase] therapy) for CPPE patients was evaluated from day 1 to day 5 after pigtail drainage by (a) vital signs; (b) complete blood count; (c) CXR; and (d) volume of effusion drained. The ultimate outcome was determined on day 5 by a physician who was blinded to clinical information of the patients based on following criteria:

*Medical success* was defined as clinical improvement as evidenced by

1. well control of infection with absence of any two of following conditions of sepsis

syndrome [S1]:
-- body temperature < 36 °C or > 38 °C
-- heart rate > 90/min
-- respiratory rate > 20/min
-- white blood cell count < 4000 or > 12000 cells/mm^3^ or > 10% immature (band)
 forms; and

1. more than 50% reduction in pleural opacity on CXR.
    .

*Medical failure* was defined as poor clinical response by the presence of

1. ongoing or progressive sepsis syndrome [S1], and
2. less than 50% reduction in pleural opacity on CXR.

**Reference**

S1. Bone RC, Balk RA, Cerra FB, Dellinger RP, Fein AM, et al. (1992) Definitions for sepsis and organ failure and guidelines for the use of innovative therapies in sepsis. The ACCP/SCCM Consensus Conference Committee. American College of Chest Physicians/Society of Critical Care Medicine. Chest 101: 1644-1655
